# Capsaicin Synthesis Requires *in Situ* Phenylalanine and Valine Formation in *in Vitro* Maintained Placentas from *Capsicum chinense*

**DOI:** 10.3390/molecules21060799

**Published:** 2016-06-21

**Authors:** Fray M. Baas-Espinola, Lizbeth A. Castro-Concha, Felipe A. Vázquez-Flota, María L. Miranda-Ham

**Affiliations:** Unidad de Bioquímica y Biología Molecular de Plantas, Centro de Investigación Científica de Yucatán, A.C., Calle 43 # 130, Chuburná de Hidalgo, Mérida, Yucatán 97200, Mexico; segarfield_88@hotmail.com (F.M.B.-E.); liarcaco@cicy.mx (L.A.C.-C.); felipe@cicy.mx (F.A.V.-F.)

**Keywords:** Solanaceae, Habanero pepper, inhibitors, chlorsulfuron, p-fluorophenylalanine

## Abstract

Capsaicinoids (CAP) are nitrogenous metabolites formed from valine (Val) and phenylalanine (Phe) in the placentas of hot *Capsicum* genotypes. Placentas of Habanero peppers can incorporate inorganic nitrogen into amino acids and have the ability to secure the availability of the required amino acids for CAP biosynthesis. In order to determine the participation of the placental tissue as a supplier of these amino acids, the effects of blocking the synthesis of Val and Phe by using specific enzyme inhibitors were analyzed. Isolated placentas maintained *in vitro* were used to rule out external sources′ participation. Blocking Phe synthesis, through the inhibition of arogenate dehydratase, significantly decreased CAP accumulation suggesting that at least part of Phe required in this process has to be produced *in situ*. Chlorsulfuron inhibition of acetolactate synthase, involved in Val synthesis, decreased not only Val accumulation but also that of CAP, pointing out that the requirement for this amino acid can also be fulfilled by this tissue. The presented data demonstrates that CAP accumulation in *in vitro* maintained placentas can be accomplished through the *in situ* availability of Val and Phe and suggests that the synthesis of the fatty acid chain moiety may be a limiting factor in the biosynthesis of these alkaloids.

## 1. Introduction

Chili plants (*Capsicum* spp.) have long been valued worldwide as an important source of bioactive compounds that are beneficial to human health. Their fruits are known not only as an important source of vitamins, carotenoids and phenolic compounds, all of which possess important antioxidant properties, but also for their pungency [1,2].

**Figure 1:**
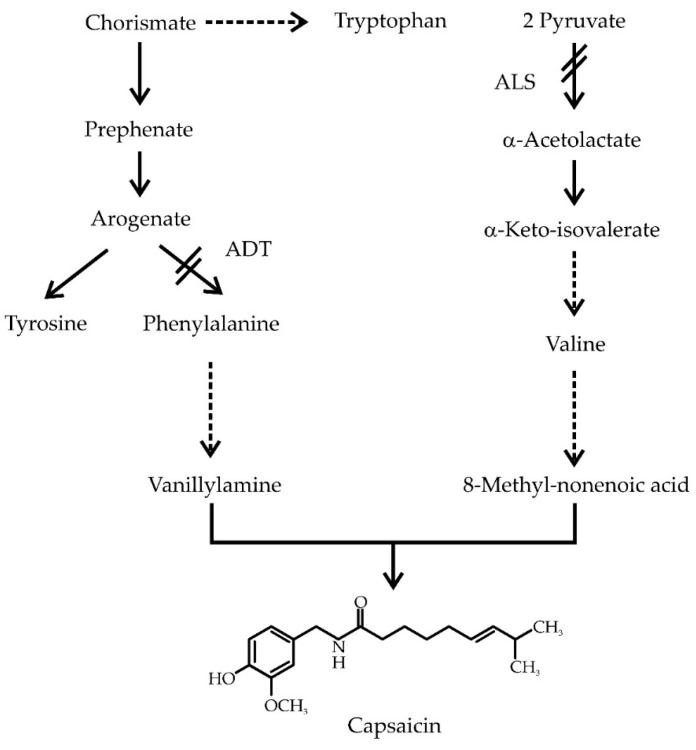
Schematic representation of phenylalanine (Phe) and valine (Val) biosynthetic pathways and their relation to capsaicinoids (CAP) synthesis.

Capsaicinoids (CAP), the pungent principle of peppers, are exclusively produced and accumulated in placentas of the hot genotypes of peppers (*Capsicum*). The ability to synthesize CAP is related to the presence of at least one functional acyltransferase allele (*AT3*) at the *Pun1* locus [3]. Recently, capsaicinoid synthase (CS) activity has been ascribed to AT3 [4]. Non-pungent peppers carry a non-functional allele, which lacks a 2.5 Kb region at the 5’ end, bearing part of the promotor and the first 735 bp of the open reading frame [5]. Although the qualitative component of pungency is controlled by *Pun1* (formerly known as locus *C*; [3,5]), the basis for the quantitative component of this trait is less known. Wide variations in the amounts of CAP produced by the different hot pepper genotypes occur [6], and even in highly isogenic lines, important variations might arise, depending on environmental conditions and pod position in the stem [7]. This suggests that the expression of CAP biosynthetic machinery can be modulated by external factors. CAP synthesis initiates from the amino acids phenylalanine and valine, from which the phenolic and acyl moieties are formed, respectively (Figure 1). Being a nitrogenous metabolite, nitrogen availability affects CAP accumulation in pepper pods [8]. Moreover, these effects are related to nitrogen availability in the placenta, the tissue responsible of CAP biosynthesis. An increase in the availability of nitrate in this tissue promoted their accumulation [9,10]. Interestingly, salicylic acid (SA) and methyl jasmonate (MeJa) induction of CAP accumulation in *in vitro* cultured pepper placentas required the activation of primary nitrogen assimilation, through the glutamine synthetase/glutamate synthase (GS/GOGAT) cycle [11]. In this way, it is clear that besides its biosynthetic capacity for CAP, pepper placentas also display the ability to secure to some extent the availability of the amino acids required in such a process. In order to determine the participation of the placental tissue as a supplier of these amino acids, herein we have analyzed the effects of blocking the synthesis of Val and Phe by using specific enzyme inhibitors. Hence, to rule out the possible participation of external sources, isolated placental tissues were maintained *in vitro*.

It has been demonstrated that both amino acids have been shown to be required to increase CAP synthesis in non-differentiated cell cultures [12,13]. However, under normal conditions, both Phe (2–22 nmol·g^−1^ FW; [14]) and Val (2–9 nmol·g^−1^ FW; [14]) cell pools in *C. chinense* placentas are very low, suggesting that their synthesis must be triggered only when it is required to satisfy specific demands [14].

## 2. Results

### 2.1. Pepper Placentas Are Able to Synthesize both Phe and Val in Situ

**Figure 2:**
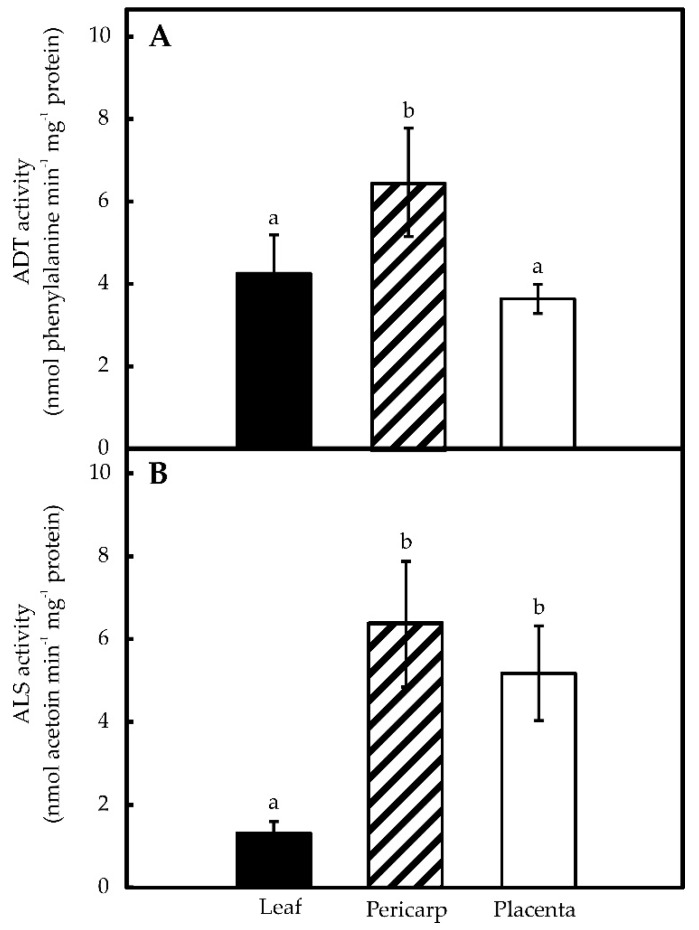
Arogenate dehydratase (ADT) (**A**) and acetolactate synthase (ALS) (**B**) specific activities in leaves, pericarps and placentas from *C. chinense*. Data represent mean ± standard deviation (S.D.) of three independent experiments with three replicates. Bars labeled with different letters are significantly different (Tukey’s test, α = 0.05).

Acetolactate synthase (ALS) represents the first committed step in the synthesis of Val, whereas arogenate dehydratase (ADT), directly channels arogenate towards Phe formation (Figure 1). Both enzymes have been shown to play critical roles in controlling the synthesis of each amino acid and were chosen as markers for the functionality of the corresponding pathways. Three different pepper tissues (leaves, pericarp and placentas) were analyzed for both ALS and ADT enzyme activities. ADT showed values between 4.0 and 6.2 nmol phenylalanine min^−1^·mg^−1^ protein, with pericarps displaying the highest activities, about 2-fold higher than that found in placentas (Figure 2A). ALS activity levels were in a similar range (*i.e.*, between 1.6 and 6.4 nmol acetoin produced min^−1^·mg^−1^ protein), with pericarps and placentas presenting much higher activities over leaves (Figure 2B). In both cases, enzyme activity values were similar to previous reports from different plant species, such as tomato, petunia and pea [15,16,17,18,19]. This is the first report of these enzymes being detected in Habanero pepper tissues, and thus, confirming Mazourek’s metabolic model for capsaicinoids synthesis, based on bioinformatic data [20].

### 2.2. Phe and Val Accumulation in Pepper Placentas Are Sensitive to p-Fluorophenylalanine (p-FP) and Chlorsulfuron (Cln)

**Figure 3:**
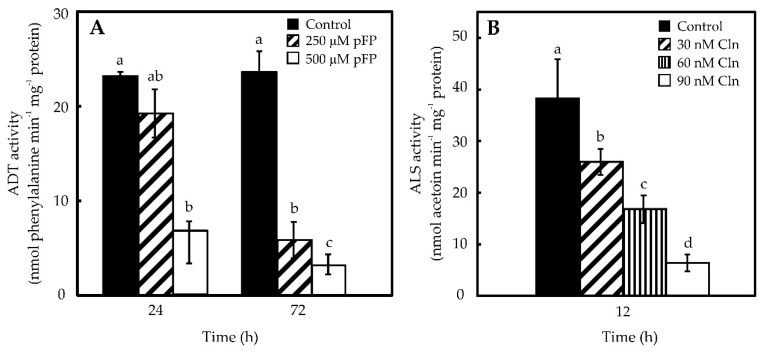
Inhibition of ADT (**A**) and ALS (**B**) specific activities in *in vitro* maintained placentas. Data represent mean ± standard deviation (S.D.) of three independent experiments with three replicates. Bars labeled with different letters are significantly different (Tukey’s test, α = 0.05).

Once the ability of placentas to synthesize Phe and Val was established, their requirement for CAP synthesis was analyzed by blocking their formation through the use of p-FP and Cln, for the inhibition of ADT (Phe) and ALS (Val), respectively. Maximal ADT inhibition (86%) was attained using 500 μM of p-FP for 72 h on placentas cultured *in vitro* (Figure 3A). Maximal ALS inhibition in placentas (84%) was obtained with 90 nM Cln after 12 h of exposure (Figure 3B). Higher doses (120 nM) and longer exposures (18 h) did not result in any further inhibition (data not shown). In this way, pepper ALS showed sensitivity to Cln similar to that from pea [15]. It should be mentioned that neither Cln, nor p-FP displayed reciprocal inhibition on each other targets (Figure S1). Furthermore, viability of the placentas, estimated as the exclusion of a vital dye (Evans’ blue), was not affected by the inhibitors’ treatments (Figure S2).

### 2.3. Blocking Phe and Val Synthesis Reduces CAP Accumulation in Pepper Placentas

**Figure 4:**
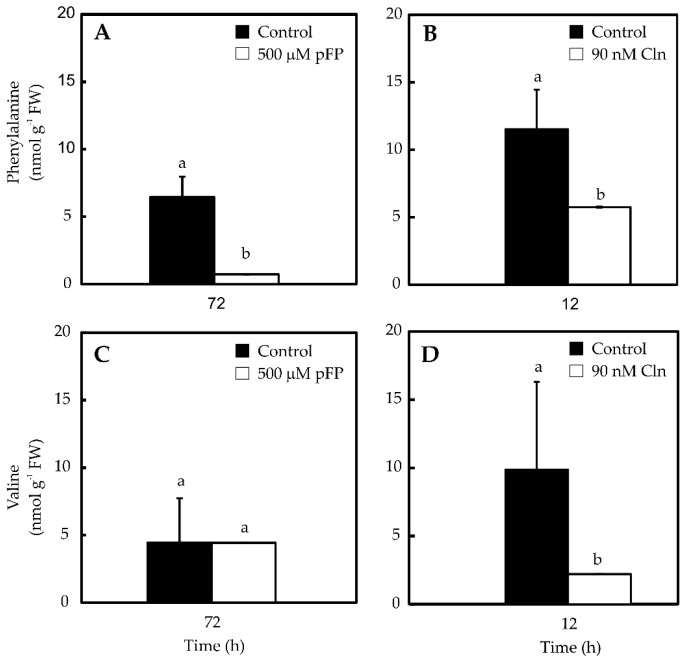
Phe (**A** and **B**) and Val (**C** and **D**) contents in placentas exposed to p-FP (**A** and **C**) and Cln (**B** and **D**). Data represent mean ± standard deviation (S.D.) of three independent experiments with three replicates. Bars labeled with different letters are significantly different (Tukey’s test, α = 0.05).

**Figure 5:**
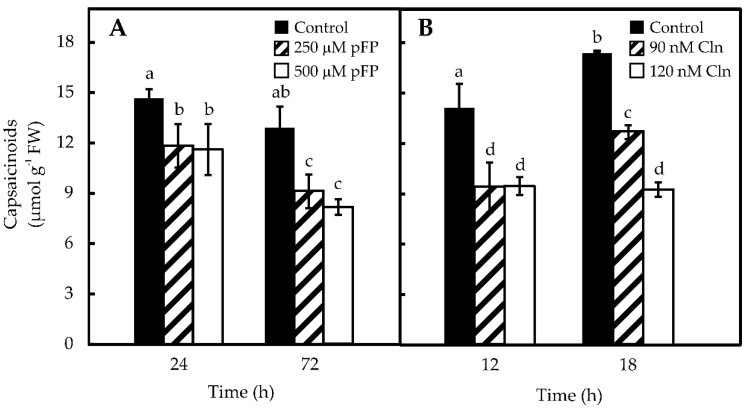
Effect of inhibition of Phe (**A**) and Val (**B**) synthesis on CAP contents in pepper placentas maintained *in vitro*. Data represent mean ± standard deviation (S.D.) of three independent experiments with three replicates. Bars labeled with different letters are significantly different (Tukey’s test, α = 0.05).

ADT inhibition (Figure 4A) practically annulled Phe pool, presenting no effect over Val accumulation (Figure 4C). On the other hand, the inhibition of ADT provoked an 80% decrease in Val content (Figure 4D), but also a *ca*. 50% decrease in Phe contents (Figure 4B). In this way, the blockage of the selected enzymes effectively reduced the accumulation of both amino acids. Correspondently, CAP contents were also reduced by the inhibitors’ treatments, although differential effects could be observed (Figure 5), since inhibition of Phe synthesis (500 mM p-FP for 72 h) only lowered CAP levels by 37% (Figure 5A), whereas Val inhibition (Cln 90 nM for 12 h) decreased CAP content up to 50% (Figure 5B). No further effects were noted by increasing neither the inhibitor doses, nor the exposure times (data not shown). In this way, locking up Phe and Val availability resulted in reduced CAP formation.

**Figure 6:**
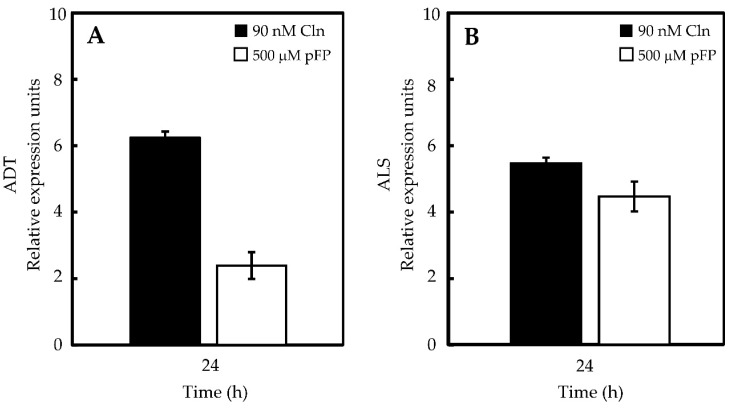
mRNA relative abundance of *ADT* (**A**) and *ALS* (**B**) in placentas maintained *in vitro*. Gene transcripts were quantified using the cycle threshold value (Ct), using actin as a reference.

The relative abundance of the transcripts corresponding to the enzymes under study was also analyzed in the placentas treated with the inhibitors. Interestingly, hampering the synthesis of both amino acids led to an increase in the corresponding transcript levels (Figure 6). However, differences in the extent of these effects were noticed, *i.e.*, inhibition of Val synthesis increased transcript accumulation for both *ADT* and *ALS*, although in a more pronounced fashion for *ADT* (Figure 6A); in contrast, the use of either inhibitor augmented *ALS* mRNAs in a similar fashion (Figure 6B).

All together, these data suggest that CAP accumulation in isolated placentas responded to decreases in the internal availability of the required amino acids, suggesting that a coordinate mechanism channeling them towards CAP synthesis is operating.

## 3. Discussion

The committed reactions for the synthesis of Phe, tyrosine and tryptophan initiate with the central intermediary chorismate and a strict regulatory mechanism controls the balance among them (Figure 1). Arogenate dehydratase (ADT; EC 4.2.1.91), which catalyzes the last step in Phe synthesis, plays a major role in this mechanism [19] (Figure 1). On the other hand, the synthesis of the branched amino acids, Val included, requires a block of four common enzymes, with acetolactate synthase (ALS; EC 2.2.1.6), having a critical participation in the regulation of the process (Figure 1).

The capacity of hot pepper genotypes to synthesize CAP is genetically controlled at the *Pun1* locus, which behaves as a single dominant and epistatic trait [3]. Nevertheless, CAP accumulation is affected by environmental and developmental factors [21,22]. In this way, variations in CAP contents can be ascribed to both genetic factors and plants’ interactions with their environment. Moreover, CAP levels may also diminish, due to chemical hydrolysis, photo-oxidation [23], enzymatic oxidation [24] or conjugation mediated by glycosyltransferases [25]. Placenta is the only tissue expressing the genes for CAP synthesis and it can convert exogenously supplied Phe and Val in CAP [26]. However, the actual origin of these amino acids, either internal or external to this tissue, has not been determined. Phe synthesis takes place in the chloroplasts of photosynthetic tissues and the possible participation of cytosol has been proposed for other tissues [19]. In fact, transcripts putatively involved in Phe metabolism have been detected in massive sequencing projects of pepper fruits [27,28]. We have shown that protein extracts from isolated placentas display ADT enzyme activity (Figure 2), which in addition was shown to be sensitive to p-FP, a specific inhibitor (Figure 3). Moreover, blocking Phe synthesis, through the inhibition of ADT, also significantly decreased CAP accumulation suggesting that at least part of Phe required in this process might have been produced internally in the placenta. Besides the phenolic moiety, derived from Phe, CAP formation requires an acyl lateral chain derived from Val. Chlorsulfuron inhibition of ALS, which initiates Val formation from pyruvate (Figure 3), also decreased Val and CAP accumulation (Figure 4 and Figure 5), thus suggesting that the requirement for this amino acid can also be fulfilled by the same tissue.

Variations in soil nitrate availability affected its own contents in placentas and directly impacted CAP contents [9]. This suggests that this tissue possess the biosynthetic potential to assimilate nitrate and then to transform it to the required amino acids: Val and Phe. This is further demonstrated by the fact that isolated placentas maintain their ability to synthesize these secondary metabolites, when cultured *in vitro* with ammonia and nitrate as sole nitrogen sources [29]. Moreover, the induction of CAP accumulation in placentas cultured *in vitro* by adding SA and MeJa required primary ammonia assimilation, through the activation of the GS/GOGAT cycle [11]. Our results show that affecting the accumulation of Val and/or that of Phe in placentas also reduces CAP accumulation. In cell suspensions, both Val [13] and Phe [30] availability have been shown to be limiting factors for CAP accumulation. However, CAP amounts in non-differentiated cultures are frequently low, perhaps due the lack of the specialized structures required for its accumulation [31]. The use of entire tissues, which preserve such structures, may represent a better model. Interestingly, when Val synthesis was inhibited, CAP content was diminished in a more pronounced fashion than by inhibiting Phe synthesis (50% *vs.* 37%), which suggests that Val synthesis could be a limiting factor in this process. The fact that by inhibiting Val synthesis, Phe content diminishes concomitantly, but that the inhibition of Phe does not affect Val content, suggests that Phe synthesis might be subordinated to the synthesis or availability of Val in placentas. Free amino acid pools are strictly controlled in plant cells and the content of one amino acid is kept in a precise balance with the others. Frequently, the internal concentration of one amino acid might control to some extent the synthesis of others, including those not biosynthetically related [32].

It has been proposed that in order to increase the production of secondary metabolites, the interface between primary and secondary metabolism could be genetically manipulated [33], so this study establishes the basis for the fundamental role of primary nitrogen metabolism in placental tissues for CAP synthesis. In this way, the use of an *in vitro* system to maintain isolated placentas has resulted in a valuable asset, since environmental conditions can be strictly monitored, so the effects due to one sole factor can be observed [34] and the use of a differentiated tissue allows obtaining data on the regulatory mechanisms closer to those that function in the whole plant, where several cell types are usually involved [31,35].

Summarizing, our data demonstrates that CAP accumulation in *in vitro* maintained placentas can be accomplished through *in situ* availability of Val and Phe and suggests that the synthesis of the fatty acid chain from Val may be a limiting factor in these alkaloids’ biosynthesis.

## 4. Materials and Methods

### 4.1. Plant Material

Habanero pepper plants (*C. chinense* Jacq.) var. Chak k’an-iik were cultivated in a greenhouse at the Centro de Investigación Científica de Yucatán (Mérida, Yucatán, México, N 21°02’38’’, W 89°38’22’’, 8 m above sea level). Plants were grown over a mixture of soil, perlite, vermiculite, peat moss and coconut fiber, and watered to full field capacity every other day. They were fertilized once a week with MaxiGro^®^ y MaxiBloon^®^ (General Hydroponics). Unripened fruits were collected from the same plant stratum (60–80 cm from soil level, plants’ average height 1.2 m), washed with commercial soap and thoroughly rinsed in running tap water. Green pods (45 × 25 mm; length × width) were collected approximately 25 days post anthesis (DPA). Placental *in vitro* system was established using five whole, isolated placentas (approx. 3 g^−1^ FW) maintained for the duration of the experiments in Erlenmeyer flasks containing 40 mL of MS medium [36] supplemented with 30 g·L^−1^ sucrose without growth regulators. Flasks were maintained at 25 °C, with continuous light and gentle shaking (80 rpm) for the duration of the inhibition experiments.

### 4.2. Inhibition of Phe and Val Synthesis

Phenylalanine and valine synthesis were blocked using p-FP and Cln, respectively. Inhibitor treatments (time and dose) were experimentally determined, applying up to 500 μM during 24 and 48 h for p-FP [30] and up to 90 nM for 12 and 18 h for Cln [37] to the placentas in the *in vitro* system described above. Cln was dissolved in tetrahydrofuran (THF) to a concentration of 12 μM and diluted in 5 mM K_2_HPO_4_ (pH 7.5), whereas p-FP was dissolved in water. Both were filter-sterilized prior to application. Samples were collected, as indicated in figures, frozen with liquid nitrogen and stored at −80 °C until analysis. Control flasks were subjected to the same manipulations, except that THF, phosphate buffer or water were added instead of the inhibitors. Samples were used to determine ADT and ALS specific activities and mRNA’s levels, as well as Phe, Val and CAP contents.

### 4.3. Determination of ADT and ALS Specific Activities

Enzyme activities were assayed in protein extracts obtained from pulverized frozen placentas. Powder was mixed with the extraction buffer (50 mM Tris-HCl, pH 8.5, 5 mM 2-mercaptoethanol and 5% (*p*/*v*) polyvinylpolypyrrolidone) in a 1:2 (*p*/*v*) ratio. The slurry was homogenized with a Polytron for 2 min and then, centrifuged at 20,400× *g* for 30 min. All manipulations were performed at 4 °C. Total protein extracts were used in the enzymatic assays. ALS activity was measured in a reaction mixture containing 20 mM phosphate buffer pH 7.0, 20 mM pyruvate, 0.5 mM thiamine, 0.5 mM MgCl_2_, 10 µM FAD^+^ and 1–5 µg total protein [15]. Resulting acetolactate was converted to acetoin by adding 50 µL 6 N H_2_SO_4_ and then quantified at 525 nm, in the presence of 500 µL 0.5% creatine and 500 µL 5% α-naphtol, prepared in 2.5 N NaOH. In order to estimate ADT activity, arogenate had to be enzymatically prepared from prephenate [38], using a partially purified prephenate aminotransferase (PAT; EC 2.6.1.78) [39] from *C. chinense* leaves. ADT activity was measured in a reaction mixture containing 50 mM Tris-HCl (pH 7.5), 2.5 mM arogenate, 0.25 mM Tyr, 1 µM leupeptin, 1 µM pepstatin, and 0.25–1 µg total protein in a final volume of 200 µL [40]. The mixture was incubated for one minute at 32 °C and then added an internal standard (0.833 mM glycine) and 100 µL OPA (54 mg OPA dissolved in 1 mL ethanol, 9 mL 0.4 M sodium borate pH 9.4 and 200 µL 2-mercaptoethanol). The reaction product (Phe) was derivatized for 90 s at room temperature, and then injected to an HPLC coupled with a fluorescence detector. Mobile phase was water–methanol (2:3; *v*/*v*), flux: 1 mL/min and running time: 6 min. Derivative reaction products were detected at 360 nm (excitation) and 455 nm (emission). The range for the Phe standard curve was 0–100 nmol. ALS activity was defined as nmol acetoin min^−1^·mg^−1^ protein, whereas ADT activity was expressed as nmol Phe min^−1^·mg^−1^ protein. Protein content was determined according to Peterson [41].

### 4.4. Determination of Metabolites in Placental Tissues

Phe and Val were separated and quantified by HPLC, as OPA-derivatives. CAP were quantified in placentas, as well as in the culture media, according to Collins *et al.* [42].

### 4.5. ADT and ALS mRNAs’ Relative Abundance

Total RNA was extracted using the method described by Rubio-Piña and Vázquez-Flota [43]. Primers’ sequences were designed according to ADT (EU616545) and ALS (EU616547) reported NCBI sequences from *Capsicum annuum* [20]. For ADT primer sequences were: forward 5′-AACCAATAATTCCACGAACCG-3′, reverse 5′-CATTTGCATCGTCCACTAGCC-3′ and for ALS, forward 5′-TGCCATCCCTCCACAATATGCT-3′, reverse 5′-ATTCTTCTGCCACCTTAGCCTC-3′. Samples were treated with DNAse I (Ambion) and cDNA was synthetized with AMV reverse transcriptase (Applied Biosystems). RT-PCR was performed using SuperScript^®^ III One-Step RT-PCR with Platinum^®^ Taq. The reaction mixture included in final volume of 20 μL:10 μL 2× SYBR Green PCR Master Mix (1×), 600 nM forward primer (0.6 μL), 600 nM reverse primer (0.6 μL), 75 ng RNA (~2 μL) and 6.8 μL RNAse-free water. RT-PCR conditions were 10 min, 95 °C, 40 cycles: (a) denaturation: 15 seg, 95 °C; (b) aligment/extension: 1 min, 60 °C (for ADT and ALS; 55 °C for actin) and (c) extension: 72 °C, 30 seg. Control samples were used to estimate differences in gene expression levels, according to the equation 2^−ΔΔC_T_^ [44].

### 4.6. Data Analysis

All of the data were subjected to analysis of variance (ANOVA), and mean comparisons were made using Tukey’s multiple-range test at a 5% level of probability.

## Supplementary Materials

Supplementary materials can be accessed at: http://www.mdpi.com/1420-3049/21/6/799/s1.
