# Supplementary material for: Capsaicin Synthesis Requires in Situ Phenylalanine and Valine Formation in in Vitro Maintained Placentas from Capsicum chinense
Source: Molecules. 2016 Jun 21;21(6):799. doi: 10.3390/molecules21060799 (PMC6273288; doi:10.3390/molecules21060799)
Supplement: Supplementary file 1 [file molecules-21-00799-s001.pdf]

Supplementary Materials: Capsaicin Synthesis Requires *in situ* Phenylalanine and Valine Formation in *in vitro* Maintained Placentas from *Capsicum chinense*

Fray M. Baas-Espinola, Lizbeth A. Castro-Concha, Felipe A. Vázquez-Flota and María L. Miranda-Ham

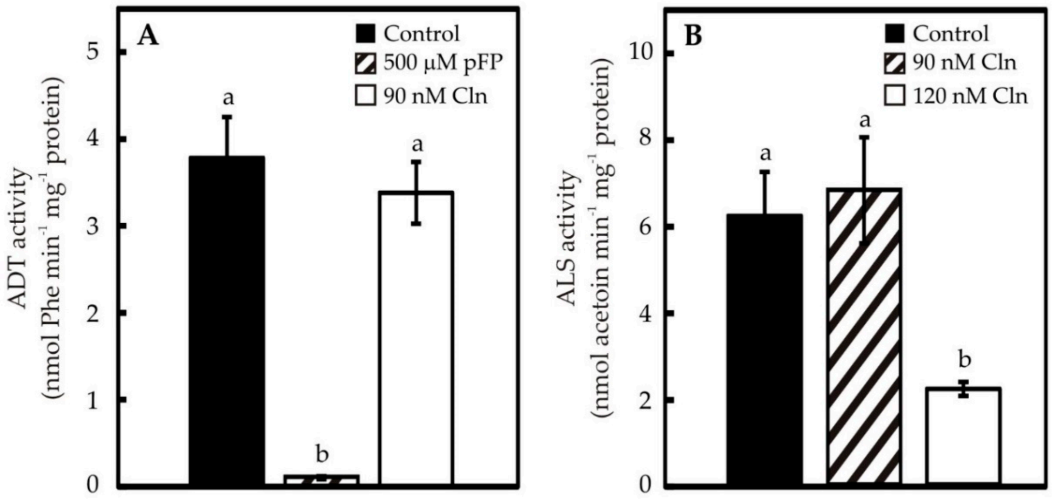

Figure S1. ADT (**A**) and ALS (**B**) specific activities in placentas treated with either p-FP or Cln to demonstrate inhibitors' specificities. Bars labeled with different letters are significantly different (Tukey's test, $\alpha = 0.05$).

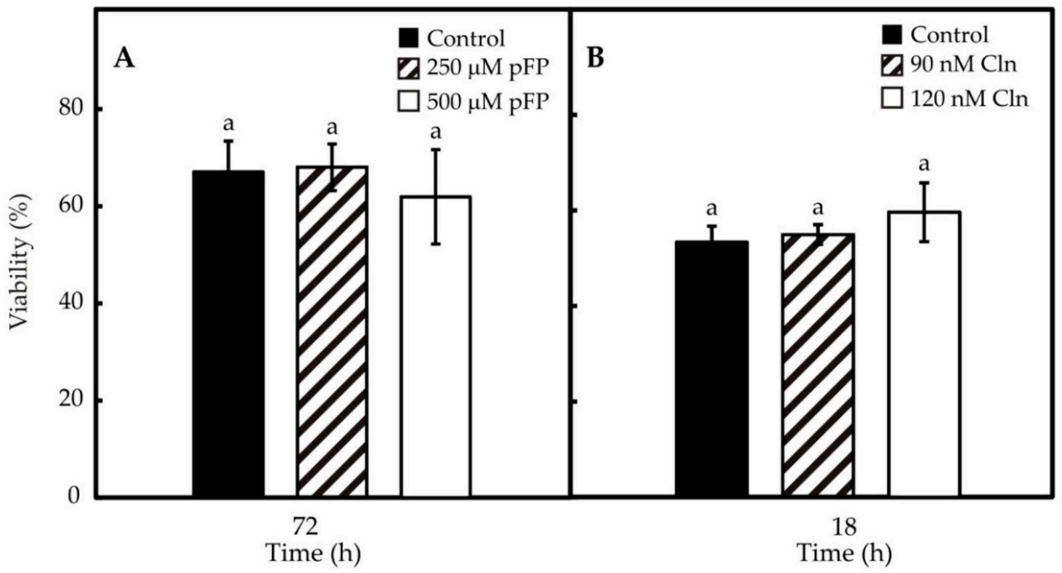

Figure S2. Viability of *in vitro* maintained placentas that were subjected to the inhibition treatments. (**A**) p-FP and (**B**) Cln. Bars labeled with different letters are significantly different (Tukey's test, $\alpha = 0.05$).
